# Detection and Classification of Alzheimer’s Disease Using Deep and Machine Learning

**DOI:** 10.3390/tomography12010004

**Published:** 2025-12-26

**Authors:** Muhammad Zaeem Khalid, Nida Iqbal, Babar Ali, Jawwad Sami Ur Rahman, Saman Iqbal, Lama Almudaimeegh, Zuhal Y. Hamd, Awadia Gareeballah

**Affiliations:** 1 Department of Biomedical Engineering, University of Engineering and Technology Lahore, New Campus, Lahore 54000, Pakistan; zaeemkhalid7864@gmail.com; 2 University Institute of Radiological Sciences and Medical Imaging Technology, The University of Lahore, Lahore 54000, Pakistan; babarali0741@gmail.com; 3 Department of Biomedical Engineering, Riphah International University, Islamabad 45210, Pakistan; jawwad.sami@riphah.edu.pk; 4 Department of Physics, University of the Punjab, Lahore 54000, Pakistan; samaniqbal.physics@pu.edu.pk; 5 Department of Internal Medicine, College of Medicine, Princess Nourah bint Abdulrahman University, Riyadh 11671, Saudi Arabia; 6 Department of Radiological Sciences, College of Health and Rehabilitation Sciences, Princess Nourah bint Abdulrahman University, P.O. Box 84428, Riyadh 11671, Saudi Arabia; zyhamd@pnu.edu.sa; 7 Department of Diagnostic Radiology, College of Applied Medical Sciences, Taibah University, Al-Madinah Al-Monawara 42353, Saudi Arabia; agsali@taibahu.edu.sa

**Keywords:** Alzheimer’s disease, machine learning, deep learning, explainability AI, magnetic resonance imaging, clinical datasets, Grad-CAM SHAP

## Abstract

Background/Objectives: Alzheimer’s disease is the leading cause of dementia, marked by progressive cognitive decline and a severe socioeconomic burden. Early and accurate diagnosis is crucial to enhancing patient outcomes, yet traditional clinical and imaging assessments are often limited in sensitivity, particularly at early stages. This study presents a dual-modal framework that integrates symptom-based clinical data with magnetic resonance imaging (MRI) using machine learning (ML) and deep learning (DL) models, enhanced by explainable AI (XAI). Methods: Four ML classifiers—K-Nearest Neighbors (KNN), Support Vector Machine (SVM), Decision Tree (DT), and Random Forest (RF)—were trained on demographic and clinical features. For stage-wise classification, five DL models—CNN, EfficientNetB3, DenseNet-121, ResNet-50, and MobileNetV2—were applied to MRI scans. Interpretability was incorporated through SHAP and Grad-CAM visualizations. Results: Random Forest achieves the highest accuracy of 97% on clinical data, while CNN achieves the best overall performance of 94% in MRI-based staging. SHAP and Grad-CAM were used to find clinically relevant characteristics and brain areas, including hippocampal atrophy and ventricular enlargement. Conclusions: Integrating clinical and imaging data and interpretable AI improves the accuracy and reliability of AD staging. The proposed model offers a valid and clear diagnostic route, which can assist clinicians in making timely diagnoses and adjusting individual treatment.

## Simple Summary

The early identification of Alzheimer’s disease can be challenging, which limits prompt diagnosis and management options. In this study, we offer a realistic approach that combines routinely observed clinical symptoms with brain imaging to detect and stage Alzheimer’s disease. The approach increases accuracy and provides practical interpretation by identifying not just important early warning indications but also pertinent brain regions using explainable artificial intelligence approaches. The findings of this study could aid in clinical decision-making and offer a flexible framework for future research to develop more precise, clearer, and more easily accessible Alzheimer’s detection and staging techniques.

## 1. Introduction

Alzheimer’s disease (AD) is the most widespread cause of dementia and develops insidiously with the selective impairment of cognitive abilities, including memory, decision-making, and communication. AD, as a common neurodegenerative disease, is considered to cause up to 70 percent of all dementia cases, especially in older people. There are a great number of diseases where forgetfulness is predominant, and dementia can be the result of various diseases [1,2]. AD is a neurodegenerative disease that is among the most common forms of dementia, leading to the loss of nerve cells in the brain and mental degeneration [3]. One of the main signs of dementia is characterized by a progressive deterioration in cognitive abilities such as memory, thinking, and communication. It is more common in the elderly, and it has a significant effect on persons who have the disease, as well as their families and caregivers. The number of cases of AD is predicted to increase dramatically as the global population ages, making it a serious public health concern.

Atrophy of the hippocampus, which plays an essential role in the formation of memories, is among the first indicators of AD. Cerebral thinning and white matter disruptions are frequently observed as AD worsens, signifying more advanced stages of the disease. These anatomical alterations in the brain emphasize how crucial early identification is to controlling and lessening the disease’s symptoms. The importance of early diagnosis is that as soon as it is identified, action can be taken; the disease can be slowed down and the quality of life can be improved, and the families can also have more time to prepare. Additionally, early diagnosis allows for the application of therapeutic measures at the most effective times, potentially delaying the onset of more incapacitating symptoms [4]. As a neurocognitive condition, dementia can take many different forms and range in severity. It includes a range of symptoms brought on by various illnesses or injuries. It interferes with a person’s everyday life by causing progressive neurodegeneration and substantial cognitive impairment. Memory loss, altered cognitive processes, behavioral issues, and deteriorating motor skills are common symptoms of dementia. Additional typical symptoms include social disengagement, emotional instability, language barriers, and a decline in drive [5].

As the world population ages, it is projected that AD cases will greatly increase in number in the next few decades. The number of people with AD is increasing because of the growing population of older people, and it is estimated that there will be a sudden increase in cases of AD. It is estimated that 6.7 million individuals aged 65 years and above are living with AD in the United States, and that this figure will more than double to 13.8 million by 2060 [6].

AD is the most common cause of dementia and leads to great morbidity and mortality. There is a build-up of beta plaques in the brain, and early amyloid markers must be tracked. AD is a leading cause of dementia, especially among the elderly. Amyloid-beta plaques and tau tangles in the brain, which cause cell and synaptic death, are indicative of the illness. The quality of life for those who have AD is greatly impacted, and family and caregivers are also heavily burdened [7,8]. The amyloid PET and MRI in vivo amyloid and anatomical markers are investigated with the help of measurements [6]. Although recent clinical trials show promise in reducing the effect of these plaques on neurodegeneration and cognition, the low amyloid burden points to the complicated and unresolved relations between amyloid PET, MRI, and cognition. Brain imaging techniques enhanced with efficient AI algorithms have emerged as a promising research pillar in cognitive diseases and have helped drug discovery, diagnosis, and prevention. Longitudinal MRI studies have shown that, when Alzheimer’s disease advances, alterations occur in the structure of the brain. Degenerative hippocampal and cortical regions have been observed in brain imaging studies over time, and these alterations are linked to cognitive decline. Furthermore, follow-up research showed that early hippocampal volume loss could predict declining memory and cognitive abilities, and a temporal understanding of cognitive decline can set parameters for new biomarker-based techniques or AI-based models for early Alzheimer’s disease detection [9,10]. Current brain–behavior research has demonstrated univariate correlations between single brain regions and cognitive decline or clinical severity using univariate approaches, which only present information in one area at a time [11].

**Figure 1:**
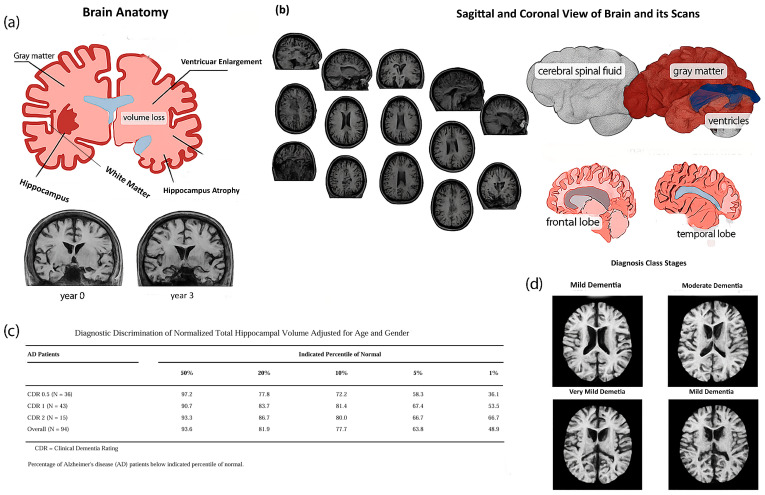
Imaging and anatomical characteristics of Alzheimer’s. (**a**) Characteristic signs include brain hippocampal atrophy and widening of the ventricles. (**b**) MRI of the brain in many views (sagittal and coronal), marked with identified areas (hippocampus, cortex, and ventricles) [12]. (**c**) Diagnostic discrimination table of hippocampal volume changes by CDR [13,14]. (**d**) MRI scans with annotations of Alzheimer stages of dementia: mild, moderate, very mild, and no dementia.

Brain structure anatomy, hippocampal atrophy, cortical thinning, and ventricular enlargement at different stages of Alzheimer’s disease, as seen in MRI scans, are depicted in Figure 1, which shows the progression of anatomical changes.

Figure 1 includes a summary of structural brain alterations and MRI scans with relevance to the diagnosis of Alzheimer’s, with volume-based diagnostics, such as hippocampal atrophy, playing an essential role in informing AI-based classification algorithms. Although CNNs, among other AI models, have been promising in detecting AD, their rational explanations are central in the clinical application of these models. Lack of transparency can make clinicians unwilling to place trust in these models, particularly where the life of a patient is concerned. Nonetheless, these approaches have some challenges since they are subjective in their interpretation, and they are not readily available, particularly in underprivileged locations [15]. For superior patient outcomes and to slow the disease’s course, early detection of AD is crucial. Improved symptom management and the best possible treatment strategies are made possible by early intervention. However, due to the subtle onset of symptoms in the early stages, early diagnosis of AD remains challenging. Therefore, developing accurate, non-invasive, and early diagnostic techniques is essential [16,17]. Moreover, early diagnosis is urgently required because it means that the disease can be treated and managed in time. Although machine learning (ML) and deep learning (DL) approaches have shown potential in the automated diagnosis of AD, a significant number of currently developed models can be described as black boxes, where the reasoning behind the predictions is not explicit, which lowers their applicability in the eyes of clinicians [18]. Delaying the progression of AD and ensuring appropriate care depend on early identification. Early identification makes early intervention possible, which can significantly improve patients’ quality of life and lessen the strain on caregivers [19,20]. As it allows for comprehensive structural images of the brain, magnetic resonance imaging (MRI) has also proven to be a helpful diagnostic technique in AD. One of the earliest signs of AD is atrophy in certain parts of the brain, such as the hippocampus, which can be detected with MRI scanning. Cognitive decline is frequently accompanied by symptoms including confusion, disorientation, and memory loss. Although MRI still requires human interpretation by radiologists, its use in conjunction with clinical symptoms improves the diagnostic specificity of AD.

Among traditional diagnostic techniques, one of the main imaging tools used to diagnose Alzheimer’s disease is magnetic resonance imaging, or MRI. MRI aids in the detection of anatomical changes in the brain that are indicative of AD, such as the hippocampus and other cortical atrophy. Memory loss and cognitive decline are examples of symptoms that typically appear after extensive brain changes have taken place [21,22]. They are no longer useful due to the increasing amount and complexity of neuroimaging data. By removing difficult-to-identify features from enormous volumes of data, deep learning (DL) and machine learning (ML) techniques have shown great promise in automating and improving AD classification. However, the majority of ML models’ black-box nature has limited their use in medical settings. With their ability to search large databases and identify complex patterns, machine learning (ML) and deep learning (DL) techniques have emerged as the leading tools for classifying AD. In clinical practice, where interpretability is crucial, explainable AI (XAI) is essential for providing transparency and trust in these models [23,24].

To address this, explainable artificial intelligence (XAI) provides concise and intelligible justifications for the model’s decisions. In order to overcome these shortcomings, explainable AI (XAI) has recently become popular, promising to provide a solution, making the decisions of the model explainable and interpretable to clinicians. With XAI methods such as Shapley additive explanations (SHAP), researchers have started to gain practical information on the contribution of each feature to an AI model making a particular prediction, thus improving clinical decision-making [25,26].

In this paper, we will seek to study how explainable AI can be integrated into the diagnosis of Alzheimer’s, specifically the concept of fusing MRI and clinical features in a binary and multi-class classification problem, such as mild dementia, moderate dementia, very mild dementia, and normal. The goal of this paper is to examine how deep learning models in MRI and clinical data can be combined to predict Alzheimer’s disease and its stages: moderate, mild, very mild, and no dementia. The focus is on using explainable AI techniques to make model output more interpretable so that it may be used more effectively in clinical contexts. Additionally, the study attempts to bridge the gap between traditional and AI-based approaches to AD diagnosis. In order to improve the interpretability and robustness of the results, this project will investigate the use of explainable AI techniques in conjunction with deep learning and machine learning models for the staging categorization of Alzheimer’s disease in the context of MRI data.

## 2. Literature Review

The use of machine learning (ML) in the diagnosis of Alzheimer’s has evolved greatly over the last ten years. Previous AI was largely built using classical statistical techniques, whereas it has since revolutionized the field. Although deep learning based on Convolutional Neural Networks (CNNs) has been successfully used to process MRI data, the potential of such models to identify the slight alterations in the internal architecture of the brain in the first phases of AD has not been investigated. It can be expected that using hybrid models that are based on multiple sources of data can further improve accuracy [27]. These machine learning techniques are especially skilled at identifying structural brain alterations that are early but not yet causing clinical symptoms. Nevertheless, a significant number of these models pay much attention to only neuroimaging data, discriminating significant clinical characteristics like patient symptoms and genetic determinants. Clinical data that has proved to be important in the detection of Alzheimer’s using machine learning includes factors like age, memory complaints, MMSE, family history, and disorientation. Another important non-modifiable risk factor is age, as the incidence of AD doubles with every five years after the age of 65. Memory impairment, mostly apparent in short-term memory, will be among the indicators of hippocampal degeneration before clinical diagnosis. Cognitive assessments such as MMSE (Mini-Mental State Examination) are pertinent, as scores below 24 correspond to cognitive decline in AD stages. A family history predisposes a person to AD, more so when the APOE4 gene is involved. The majority of cases of disorientation in time, place, and identity are moderate and can be easily identified in concert with parietal/temporal lobe impairment. The inclusion of these factors in ML models also helps distinguish normal aging, MCI, and AD better, which can lead to a faster diagnosis and treatment [28,29,30].

Many studies have focused on building automated AD classifiers with mainly neuroimaging data, including Positron Emission Tomography (PET) and MRI scans. Traditionally, characteristics from various imaging modalities have been extracted using machine learning (ML) techniques to differentiate between AD, mild cognitive impairment (MCI), and normal control (NC) groups. Early research relied on simple categorization algorithms, while more recent developments have included more sophisticated methods, like deep neural networks (DNNs) [31,32].

When diagnosing AD clinically, MRI is often the first modality used. It can produce high-resolution pictures that show signs of atrophy and other structural alterations in the brain that are characteristic of AD. Imaging is usually used to support symptom-based diagnosis, and imaging results are validated by clinical history and cognitive tests. However, using these traditional methods exclusively often results in late-stage diagnosis [33].

Multiple approaches based on brain imaging biomarkers like MRI have been used in previous studies to diagnose Alzheimer’s disease using a range of AI and ML models. In order to differentiate between various phases of AD, these researchers typically use neural networks, support vector machines (SVMs), and other classifiers.

The assessment of brain anatomy and the identification of anomalies linked to AD are two more common uses for MRI. To increase classification accuracy, imaging data is combined with symptom information, such as cognitive tests (like the Mini-Mental State Examination). Convolutional Neural Networks (CNNs), one type of deep learning model, have been proposed for the early diagnosis of AD from MRI images. Mild cognitive impairment (MCI), which is regarded as a prodromal stage of AD, is one of the several phases of AD that these designs are highly successful at classifying [34]. Convolutional Neural Networks (CNNs), a type of deep learning technique, have demonstrated exceptional performance in identifying medical images for the identification of AD. Without the need for human feature extraction, these models can automatically learn features from raw PET and MRI data. Furthermore, to increase classification accuracy, previous research has used hybrid approaches that blend ML and deep learning models with symptom data. Hippocampal atrophy, cortical thinning, and ventriculomegaly can be extracted by deep learning models trained on volumetric MRI data and are available as a feature automatically. These structural characteristics are graphically illustrated in Figure 1, where the role of MRI data in multi-stage AD classification should be emphasized. These neuroanatomical alterations are associated with the development of AD from a very mild to a moderate stage. Decreases in the size of the hippocampus can be observed at the initial stages, and the further stages feature significant ventricular widening and cortical atrophy. Such patterns can help deep learning models to distinguish between healthy aging, MCI, and AD, providing the possibility of a scalable and objective staging of diseases [12,35,36].

Encouraging results have been obtained recently with multimodal methods combining both neuroimaging information and clinical characteristics, which have been able to considerably increase classification accuracy [37]. Their performance has been advanced by adding clinical characteristics, including cognitive scores and symptom complaints, to MRI biomarkers to enhance classification accuracy. Another advancement is the connection of the MMSE score, disorientation symptoms, and factors related to age to neuroimaging, since this enables the discovery of early Alzheimer stages, as compared to using imaging or clinical variables alone. Combining structural-degeneration-based multimodal models (using MRI) and cognitive symptoms shows better sensitivity and specificity in both binary and multi-class AD classification [38,39]. For example, a hybrid CNN model with MRI scans and clinical data outperformed models that used imaging data alone in the diagnosis. Such a combination of modalities provides the possibility of more holistically considering the disease, better distinguishing between Alzheimer’s and other cognitive disorders, including mild cognitive impairment (MCI).

The diagnosis of AD has always been viewed as a binary classification issue (AD vs. non-Alzheimer’s disease). Multi-class classification models, which differentiate between MCI, early AD, and late AD, have been made possible by new technology. To guarantee the highest level of classification stability, these systems also frequently combine MRI data with clinical results such as genetic markers and cognitive test data [40]. Models for binary categorization separate NC from AD or MCI. Models for multi-class categorization differentiate between several phases of AD, such as normal, MCI, and late AD. CNNs and XGBoost are examples of deep learning models that have been successfully applied to both binary and multi-class problems.

Although the development of AI systems in Alzheimer’s diagnosis is very promising, one big problem facing the implementation of AI systems in clinical practice can be attributed to the level of incomprehensibility of most deep learning systems. Although such models perform well in terms of making their predictions, they have yet to be implemented by clinicians, as these models are often perceived as black boxes, which is especially true in high-stakes settings like healthcare. Such a lack of transparency has led to a surge in research on so-called explainable AI methods, which aim to provide insight into how an AI model arrives at its decisions. Methods like SHAP and Grad-CAM are being used on Alzheimer’s diagnostic models, giving the clinician a more explainable reason as to why a specific diagnosis was made.

Alzheimer’s disease (AD) diagnostic techniques have been developed recently, in large part due to multimodal and explainable AI frameworks. For instance, a hybrid CNN–SVM pipeline that provided interpretability based on Grad-CAM and merged MRI and cognitive variables achieved a diagnosis accuracy of approximately 92% [41]. According to that research, the Grad-CAM tool’s explainability enables quicker and better decision-making. Additionally, feature-level explainability improves diagnostic modeling transparency and builds clinician trust [2]. There are still difficulties and barriers in spite of these improvements in study designs and resources. The interpretability and generalizability of models are still somewhat constrained by limited availability to multimodal datasets with quality annotations, the process of putting technological designs into practice, and access to different multimodal sources (such as MRI, cognitive screening, and clinical measures) [24]. According to some more recent studies that combine MRI-derived biomarkers with cognitive screening data, multimodal trust concerns can be integrated to create a good compromise between interpretability, knowledge preservation, and accuracy [42]. By using these methods, we may create AI models that are more transparent, clinician-relevant, and effective. We can also lay the foundation for future research on multimodal work that detects the disease and also identifies the stage of disease, and explains the feature that represents the presence of a disease.

One of the current challenges in the diagnosis of Alzheimer’s is the limited sources of annotated multimodal data, which are needed to design robust and general models. Moreover, they should develop ways of merging data collected by different modalities (MRI, genetic information, and clinical measurements) and still maintain a sense of the outcome. Although considerable ambiguity has been clarified, much remains to be accomplished to verify that the AI models of Alzheimer’s diagnosis are accurate and explainable. Future work should further concentrate on enhancing the strength of these models and dealing with the multimodal data combination issue to attain better diagnostic performance [43]. Implementing an explainable AI method, the authors recently created an integrated model that combines MRI-based feature extraction with cognitive screening data, maintaining a high degree of accuracy and interpretability. This addition highlights the continued importance of multimodal methods in Alzheimer’s diagnosis. A more comprehensive and in-depth understanding of the disease’s history and dynamics may result from combining neuroimaging biomarkers with clinical and cognitive data. Better models for diagnosis will be established with the aid of this kind of dual-modal system, which will also make the model more transparent and reliable in clinical settings. In keeping with these advancements, our study emphasizes the ongoing and complementary significance of MRI-derived feature and symptom data and demonstrates how merging both data inputs could enhance model robustness and clinical relevance in more practical clinical decision-making scenarios. While we stress that improving model reliability, generalizability, and interpretation in clinical research is the foundation of diagnostic studies, future research will focus on ways to further optimize multimodal data, then expand its use, and establish ways to integrate modes [41].

Even with the encouraging advancements, there are still several issues with using deep learning and machine learning to diagnose AD. The lack of sizable annotated datasets on which to train the models is the first of these issues. Second, because the majority of deep learning models are “black boxes,” which makes it difficult for physicians to understand them, the interpretability of these models continues to be a significant difficulty. Furthermore, using multimodal data—such as clinical, MRI, and genetic data—is difficult from a technological and computational standpoint. Despite the achievements, there are still many difficulties, especially when it comes to improving interpretability of black-box models. Most ML and DL models are criticized for being opaque, meaning that clinical practitioners find it difficult to adopt them. Additionally, there are also issues with using limited expert datasets and combining multimodal data (such as genetic, neuroimaging, and cognitive scores).

## 3. Materials and Methods

This study proposes a dual-mode diagnostic framework in which clinical symptom-related data are combined with structural neuroimaging by MRI to categorize the stages of Alzheimer’s disease (AD) with a great level of accuracy. The clinical mode displays demographic characteristics (age, gender, education), cognitive test results, memory complaints, and early disorientation—all the features that are known to be present in the early stages of AD development [44]. The MRI mode is dedicated to such structural biomarkers as hippocampal atrophy, ventricular enlargement, and cortical thinning that frequently occur before the appearance of blatant clinical signs [45]. To examine these two modes, we use classical models of ML (KNN, SVM, Decision Tree, Random Forest) on the clinical data and state-of-the-art DL models (CNN, EfficientNetB3, DenseNet-121, ResNet-50, MobileNetV2) on MRI scans for stage-wise classification. To achieve trust and interpretability of model outputs, explainable AI (XAI) can be used, such as SHAP (in the case of clinical features) and Grad-CAM (in the case of MRI images), where it is possible to see which feature and brain region are the most significant in diagnosis [46].

All model training and experiments were run with the help of Google Colab (Google LLC, Mountain View, CA, USA) and Kaggle Notebooks (Kaggle Inc., San Francisco, CA, USA) using their GPUs/TPUs and pre-installed ML/DL packages (TensorFlow (V. 2.12), PyTorch (V. 2.1), Keras (V. 2.12), etc.). Google Colab also supported deep learning model convergence, accelerated with the help of GPU/TPU, which allowed faster convergence. Kaggle was utilized in preprocessing of the datasets, exploratory data analysis, version control of notebooks, and reproducible storage of models/results. The use of these tools in recent biomedical studies on AI has also been common because they are accessible, cheap, and effective in computations, which is why they are perfect in the AD classification process using large datasets of MRI data [47,48].

**Figure 2:**
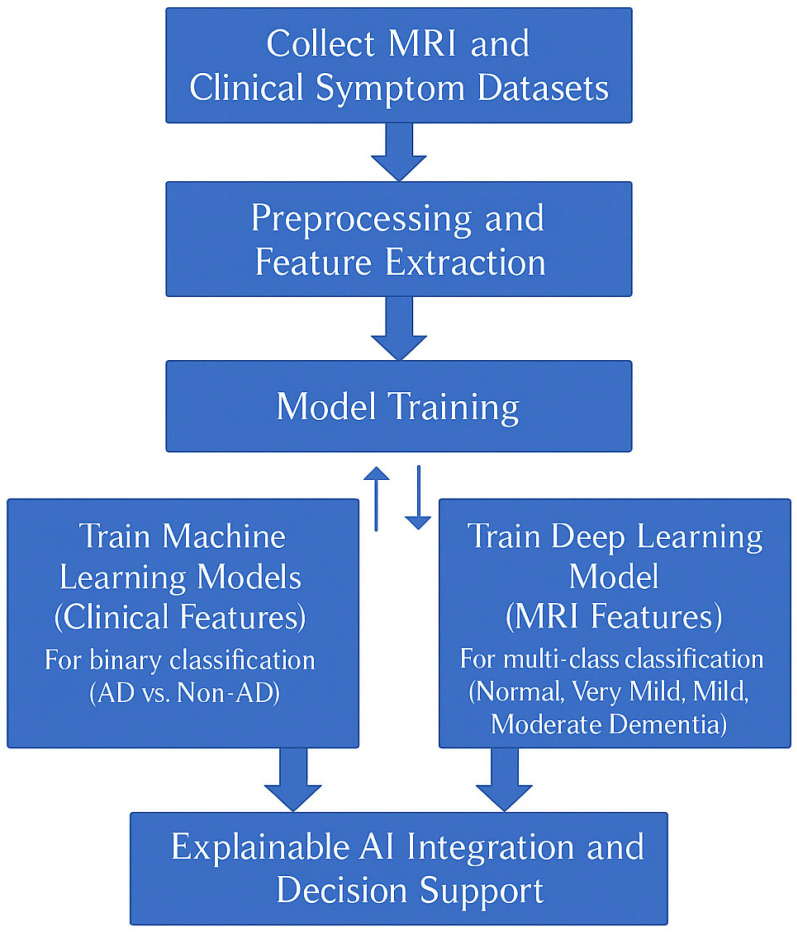
Scheme of the complete pipeline of the proposed Alzheimer classification based on the combination of MRI and clinical characteristics through machine learning and deep learning techniques.

This paper uses a dual-mode approach as graphically workflow explained in Figure 2, using stringent structural MRI scans and symptom-based clinical data to classify Alzheimer’s and its stages with a lot of confidence. The MRI data consists of high-resolution sagittal and coronal brain scans with labeling of primary anatomical structures such as the hippocampus, cortex, and ventricles, as they are considered to undergo significant degeneration in the process of Alzheimer’s development. The features were extracted as structural biomarkers, such as hippocampal atrophy, cortical thinning, and ventricular enlargement, and they are supposed to be predictive. Correspondingly, the clinical data contain information on individual features of patients (age, MMSE (Mini-Mental State Examination) scores, memory complaints, disorientation symptoms, and family history of dementia) that are major non-imaging predictors of cognitive decline. A combination of the two data sources can be used to form a comprehensive strategy by way of binary and multi-class classification. The MRI images were downloaded through the OASIS Alzheimer’s Detection dataset (https://www.kaggle.com/datasets/ninadaithal/imagesoasis, accessed on 25 September 2024), and the clinical data were downloaded through the ADD (https://www.kaggle.com/datasets/rabieelkharoua/alzheimers-disease-dataset, accessed on 25 September 2024). No fresh information was gathered specifically for this investigation. Institutional review board approval was not necessary because this work involves an earlier examination of publicly accessible datasets.

### 3.1. Dataset Description

There are 2149 total patients in the clinical dataset: 1088 males and 1061 females, and 1389 patients without dementia and 760 patients with dementia. Demographic data and cognitive traits (MMSE scores, memory complaints, disorientation symptoms, and family history of dementia) are included in every patient record. Based on CDR labeling, we classified the 86,437 brain scans in the MRI dataset into four clinical stages: normal (67,222) and very mild (13,725), mild (5002), and moderate dementia (488). To ensure uniform spatial resolution, all of the MRI scans were preprocessed for skull stripping, intensity normalization, and scaling to 128 × 128 pixels. The comprehension of the population size and distribution throughout the stages that guide the clinical and imaging evaluations is undoubtedly improved by this detailed presentation of the datasets. The methodology of this study is illustrated in the Figure 2.

### 3.2. Preprocessing

Clinical data, including age, MMSE scores, memory and disorientation complaints, and family history, were cleaned, with missing values imputed, and label-encoded where necessary. Z-score normalization was implemented in order to ensure consistency of the scale across features. The Z-score for each feature was calculated as

(1) $$z=\frac{x-\mu }{\sigma }$$

where *x* is the initial feature value, *μ* is the mean, and *σ* is the standard deviation of the feature [49].

These were processed features that were mainly used to differentiate between Alzheimer’s and non-Alzheimer’s cases. To process the MRI data, all the scans were resized, normalized in intensity, skull-stripped, and turned into grayscale slices. The main anatomical structures, such as the hippocampus, cortex, and ventricles, were enhanced through the use of preprocessing techniques, allowing for the acquisition of fine-grained spatial biomarkers. The MRI dataset showed a notable class imbalance, with significantly fewer samples in the moderate dementia category than in the other four phases. A two-step strategy was used to deal with this problem. Initially, we used manipulations (horizontal and vertical flipping, random rotation of the images (±10 degrees), modest Gaussian noise injections into the views, and brightness fluctuations (±15%)) to enhance the minority classes. We were able to add diversity to the minority classes as a result. Second, DL was trained using a class-weighted categorical cross-entropy loss term, which meant that the model suffered a higher penalty for model categorization for underrepresented classes. Any potential class imbalance and related model bias in favor of the majority (non-dementia) class are lessened by this two-pronged problem-solving approach. This imaging preprocessing pathway played a crucial role, particularly in categorizing the disease stage, including normal and very mild, mild, and moderate dementia. This two-rung preprocessing flowline optimizes the convergence of both sets of data, with the intended goal of diagnosis: symptom-based detection of the disease and staging through imaging of disease progression.

### 3.3. Machine Learning Model for AD Detection

A supervised machine learning framework was adopted based on more detailed clinical data containing initial manifestations of the disease, with the help of which it was possible to identify Alzheimer’s in the early stages. This information included a range of cognitive and demographic variables, especially age, scores of MMSE, memory and disorientation, family history, and other diagnostic factors. Those characteristics have been clinically shown to be associated with Alzheimer’s disease development and were used due to their predictive capabilities in two-category classification: Alzheimer’s disease and non-Alzheimer’s disease. To obtain informal conclusions about the dataset, an exploratory data analysis (EDA) was performed by displaying the distribution of each feature with graphical models. For this purpose, the clinical features were prepared for graphical visualization as follows.

**Figure 3:**
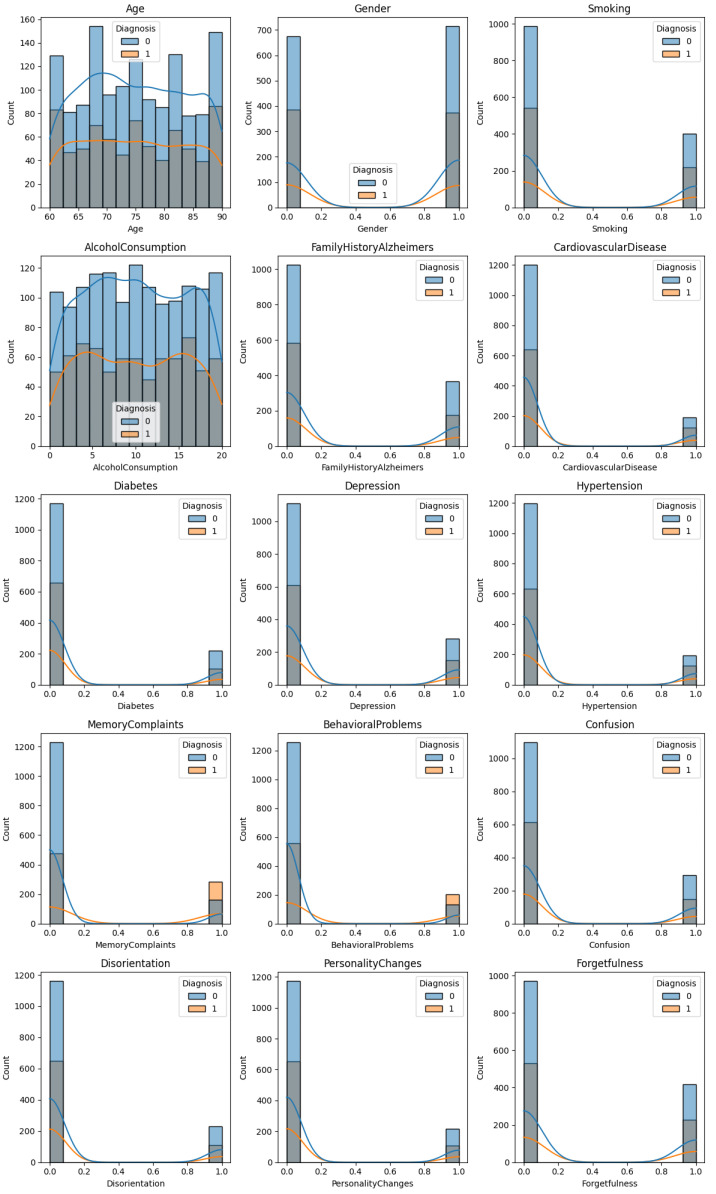
Clinical dataset graphical visualization.

**Figure 4:**
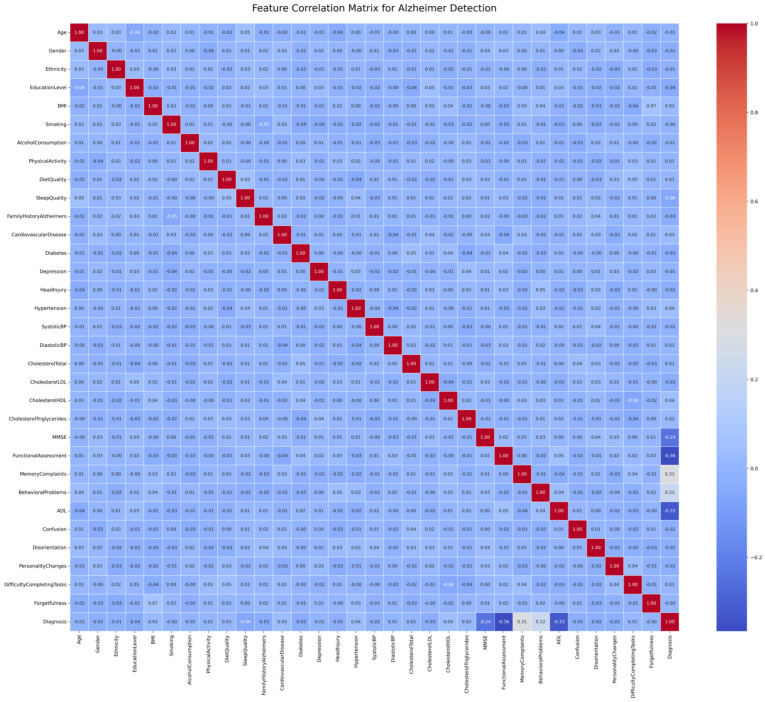
Correlation heatmap of clinical features for Alzheimer’s prediction.

The correlation heatmap, constructed with the help of the Matplotlib (V. 3.8) and Seaborn (V. 0.13) libraries, helped visualize the inter-feature relations by showing (Figure 3 and Figure 4) the correlation between symptoms and the diagnosis of Alzheimer’s disease.

In addition, heatmaps in the form of confusion matrices were employed following the model assessment to see true and false outcomes and reveal the classification performance on both Alzheimer and non-Alzheimer cases. There was a stratified split strategy to achieve the proportion of the classes; the dataset was divided into 50 percent for training and 30 percent for validation. The test data were the remaining 20 percent. This split guaranteed an equal tuning of models and objective final testing. Four well-known machine learning algorithms were implemented: K-Nearest Neighbors (KNN), Support Vector Machine (SVM), Random Forest (RF), and Decision Tree (DT). All models were trained using Z-score-normalized data to normalize the feature range and enhance convergence. The validation set was used to tune the hyperparameters so as to optimize performance without overfitting.

We chose KNN (*k* = 10). To use the local neighborhood structures in the symptom space, the k parameter was chosen as *k* = 10, depending on the validation accuracy. Compared to past studies that deployed low default values of *k*, our selection was better in terms of robustness under noisy instances and also due to simplicity. The model was effective at measuring proximity-based patterns of symptoms commonly found within early-stage AD profiles.

In our Alzheimer classification scheme, we used a linear Support Vector Machine (SVM) on Z-scored clinical symptom data to differentiate between Alzheimer patients and non-Alzheimer patients. SVM learns a sign-separating hyperplane based on the following equation:

(2) $$f\left(x\right)=W^{T}x+b$$

Here, *x* is the input feature, *W* is the weight, and *b* is the bias [50].

This hyperplane is optimized to have the maximum margin between the two classes. The margin is defined as the distance to the two hyperplanes that are closest to the support vectors (the data points that are critical for defining the decision boundary). Generally, a larger margin leads to better generalization. Although many existing studies emphasize the use of non-linear kernels (e.g., RBF), without a clear rationale for the choice, we found that linear SVM performs best on our dataset after normalization. Therefore, symptom-based features in our dataset are linearly separable. This not only simplified and sped up the training process but also improved clinical interpretability, allowing us to easily understand why a particular prediction was made, which is crucial in medical decision-making.

We used a Decision Tree model with a tuned depth of 5, aiding in balancing the performance and explanations. The model is trained to learn hierarchical decision rules based upon clinical symptoms according to their Gini impurity criterion, which is used to determine the likelihood of incorrectly identifying a randomly selected element. In contrast to the previous literature, which tolerates trees that are overgrown, our method encourages transparent and clinically interpretable choices, which can be of practical use among non-technical medical professionals.

Multiple shallow learners were aggregated with the Group approach with a Random Forest model of 100 estimators and a max depth of 5. This method of ensemble-enhanced generalization was still interpretable. Unlike most related works, which train RFs on non-preprocessed data, we have employed Z-score normalization as well as stratified splitting, both of which are known to enhance fairness and reproducibility.

We utilized SHAP to estimate both the effect of each clinical feature on the model’s output and to enhance the interpretability of our Random Forest model because we obtained the best results with this model. The Tree Explainer was used to perform SHAP calculations, maximizing ensemble models, and was thus optimized to work with Random Forest. A Z-score was applied to normalize the clinical features and label them using interpretive care. This explainability stage served as a strong basis for the visualization of which clinical variables (MMSE, brain volume, and age) had the largest predictive impact on Alzheimer’s, which was contextualized at individual and global levels.

### 3.4. Deep Learning Model for AD Stage Detection

**Figure 5:**
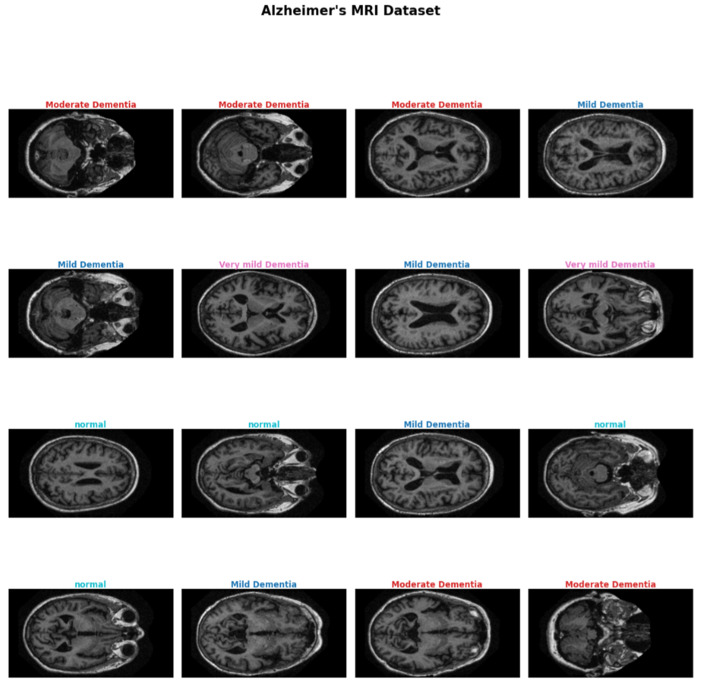
Images of brain MRI slices demonstrating the four stages of Alzheimer’s, consisting of normal, very mild dementia, mild dementia, and moderate dementia, showing atrophy of the progressive hippocampus and enlargement of the ventricles as the disease progresses.

This research provides a deep learning pipeline to carry out a stage-wise classification of AD into four stages—normal, very mild dementia, mild dementia, and moderate dementia—using MRI scans of the brain. In Figure 5, representative MRI slices depict the four classes studied, featuing the gradual structural changes; i.e., with disease progression, hippocampal atrophy, together with ventricular enlargement, becomes more pronounced. Such visual (Figure 5) discrepancies serve as the cornerstone of deep learning models to draw the line between early and late stages of AD.

In this framework, 5 rapidly developing deep learning models, CNN, EfficientNetB3, ResNet-50, DenseNet-121, and MobileNetV2, are used for precise and comprehensible classification of AD stages. Our preprocessed grayscale MRI slices underwent skull stripping, intensity normalization, and data augmentation to enhance generalization. To avoid overfitting, early stopping and dropout were included.

Five deep learning architecture models were used and compared: Custom CNN, EfficientNetB3, ResNet-50, DenseNet-121, and MobileNetV2. All of them were chosen carefully to strike a balance between accuracy, computational efficiency, and clinical relevance. ResNet-50 was used to take advantage of its deep residual learning, with skip connections that preserve flow and avoid the vanishing gradient problem in deeper networks. It has a 50-layer design that gives it both the low-level textures and high-level structural variations in the brain. In this case, the pretrained backbone was not fine-tuned; instead, a new dense head was used to perform classification. Residual connectivity in ResNet-50 enabled the model to show better stage distinction, especially distinguishing between moderate, very mild, and mild cases of dementia, a process that greatly benefited from its depth and the nature of residual connectivity, which is sensitive enough to recognize the presence of neurodegeneration.

The EfficientNetB3 model was selected based on its compound scaling technique, which provided a good compromise between the depth and width of the model and input resolution, so higher results in terms of accuracy can be obtained without making the model computationally intensive. In this work, the 50 top layers were unfrozen in order to fine-tune them to specific AD patterns. As a means of increasing generalizability, a bespoke classification head with dense and dropout layers was included. EfficientNetB3 demonstrated reliable performance in terms of high validation accuracy with fewer parameters than most conventional deep models, which opens up the possibility of a clinically viable evaluation method for high-resolution MRI-based AD four-stage screening.

DenseNet-121 was chosen due to its densely connected nature, which ensures feature reuse and good gradient flow, as the layer is exposed to all previous layers. Dropout and SoftMax layers were added to the pretrained base in the form of a custom classification block. This model achieved a high generalization capability, especially on underrepresented classes, because of its effective feature transmission and capability to emphasize the minor trends, such as early hippocampal atrophy. DenseNet-121 was put to clinical use since it was clinically effective, and on datasets in which some examples outnumbered others, it controlled overfitting, and its connection between layers contributed to more comprehensive representations.

MobileNetV2 was used to provide a model that was capable of efficient execution in resource-limited clinical settings whilst maintaining high performance. Its bottleneck layers and inverted residuals enable the model to excel at visual tasks with much less computation and memory cost as compared to conventional deep networks. MobileNetV2, in our assessment, obtained high accuracy and a very fast inference speed, and thus, it would suit a real-time or mobile-based AD screening application well. Due to its efficiency, the detection of dementia with the help of AI has the potential to become available beyond the facilities of high-end clinical practices, which is also in line with the current trend of portable and point-of-care diagnostic tools.

The baseline architecture used to classify AD stages is a Convolutional Neural Network (CNN). Though lightweight, this CNN was designed in a way that it could recognize disease-specific structural differences like hippocampal atrophy and cortical thinning, which are evident in MRI images. It has three convolutional layers, including ReLU activation, which are interlaced with max-pooling of down-sampled features, as well as dropout layers in the regularization layer procedure. The network was trained on augmented MRI data. Although the CNN structure is quite simple, it still managed to serve as an entry point in distinguishing early AD levels with the usage of well-preprocessed MRI data.

For clinical trust and transparency, the use of Gradient-weighted Class Activation Mapping (Grad-CAM) was used to visualize the areas of the brain that affected the decision of the model. The heatmaps created showed that the models paid considerable attention to the hippocampal and ventricular areas, which makes sense in terms of neurological knowledge of AD progression. This process of interpretability closes the gap between the AI predictions and the clinician’s thought process, easing the burden of decision-making around a sensitive neurodegenerative diagnosis.

### 3.5. Model Architecture and Hyperparameters

All features in the machine learning models that were trained using clinical symptom data were first standardized using Z-score scaling. KNN employed *k* = 10 to strike a compromise between sensitivity to local neighborhood structures and robustness. The best generalization was achieved by SVM using a linear kernel with C = 1.0 since the clinical features were standardized and linearly separable. In accordance with the stratified 50/30/20 train/validation/test split, the Random Forest consisted of 100 trees with max_depth = 5, while the Decision Tree was restricted to a maximum depth of 5 to preserve interpretability while avoiding overfitting. For all of our deep learning models trained on preprocessed grayscale MRI slices, the Adam optimizer was used to optimize all networks with a learning rate of 1 × 10^−4^, β_1_ = 0.9, and β_2_ = 0.999, batch size = 32, and 50 epochs, including early halting with a patience of 5 to avoid overfitting. Dropout (0.3) was used on every network. The Custom CNN included a 128-unit dense layer with dropout after three convolutional layers (3 × 3 kernels, ReLU) interspersed with max-pooling. To take advantage of residual connections for better AD stage distinction, ResNet-50 used a pretrained, frozen 50-layer residual backbone with only the classification head trained. Together with a unique dense head and dropout, EfficientNetB3’s top 50 layers were unfrozen for fine-tuning, taking advantage of compound scaling to achieve a balance between computational efficiency and model complexity. DenseNet-121 enabled feature reuse and generalization of underrepresented classes by combining a pretrained base with a bespoke classification block that included dropout and SoftMax. MobileNetV2 achieved good accuracy with quick inference and is appropriate for mobile or resource-constrained clinical applications by using bottleneck layers, inverted residuals, and a bespoke dense head. Skull-stripped, intensity-normalized, and augmented MRI slices were used by all DL models, while Z-score-normalized clinical features were employed by ML models. Based on validation performance, this empirical hyperparameter selection enables complete reproducibility.

In the proposed framework, the MRI (DL-based) and clinical (ML-based) pipelines are two separate diagnostic modules that rely on different data modalities. Based on the participants’ cognitive and demographic characteristics, the clinical mode only uses binary classification to identify Alzheimer’s vs. non-Alzheimer’s. The MRI mode performs stage-wise categorization (normal and very mild, mild, moderate dementia) using neuroimaging data. In order to preserve the interpretability and diagnostic power of each modality independently, predictions from the two branches were assessed separately in this investigation. In order to improve overall robustness and clinical value, future work will concentrate on combination/ensemble fusion by employing weighted averaging or meta-learning techniques that integrate both sets of outputs into a single joint inference system. We used a subject-wise split with no overlap between our training, validation, and test sets (50%, 30%, and 20%) to avoid data leakage. Batch normalization, early stopping (patience = 5 epochs), and dropout (0.3) were regularization strategies. Validation curves confirmed our evaluation procedure and demonstrated steady model training without overfitting. These steps ensured that the model’s capacity for generalization was appropriately reflected in the performance results and analyses that are shown in the Section 4.

Each experiment was conducted using an NVIDIA Tesla T4 GPU, 16 GB of RAM, and an Intel Xeon CPU in Google Colab Pro, Kaggle Notebooks, and local Jupyter notebook environments. For each of these platforms, batch sizes and learning rates were tuned to provide steady convergence and effective model training in spite of hardware constraints. For full disclosure of the computing resources used in this work, the following information is available.

## 4. Results and Discussion

The experimental findings in this paper present a thorough comparison between the conventional ML models and the DL model structures in the assessment of stage-by-stage classification of AD. This dual-modality structure will use clinical characteristics that depend on symptoms for first-line screening and further process neuroimaging by MRI to calculate the true stages. With this combination of complementary approaches, the proposed pipeline can achieve not only baseline predictive capacity with ML but also accuracy and a clinically interpretable model with DL. The classification was performed to identify four clinically known stages of AD, consisting of normal, very mild dementia, mild dementia, and moderate dementia. This is a multi-class problem, which is inherently hard because early-stage AD harbors subtle structural and symptomatic differences that, in many cases, overlap with healthy cases. We have demonstrated that the symptom-based ML approaches are a practical initial diagnostic tool, but the MRI-based DL frameworks are much better at detecting subtle pathological patterns and making an automatic identification that connects to later clinical manifestations.

Four traditional ML algorithms—KNN, SVM, Decision Tree (DT), and Random Forest (RF)—have been used as a starting point to detect Alzheimer’s by training them on clinical symptom information. This step is clinically important because it offers a non-invasive first-line assessment for the occurrence of Alzheimer’s prior to a confirmation using MRI. The models exhibited different levels of ability to perform multi-class classification of the four clinical stages. Random Forest and Decision Tree performed the best and obtained test accuracies of 97.50% and 96.33%, aptly describing their propensity to model the interactions of complex features. SVM obtained 84.42%, a reasonable accuracy, with KNN having a good baseline value of 72.33, which makes it clinically useful for a primary screen.

The results (Table 1) demonstrate the accuracy of the four traditional ML models used with symptom-based data as the first step of diagnosis, before the deep learning stages based on MRI information.

Next, the accuracy and validation of the ML models are reported.

Random Forest presented the highest accuracy of 97% on test data, with Decision Tree at 96%, thereby ranking the second highest in accuracy. Such high performance is attributable to the fact that they are capable of dealing with non-linear connections between clinical characteristics. SVM, with an accuracy of 84%, presented moderate classification strength, whereas KNN had an accuracy of 72%, with a simpler but clinically significant foundation to entail a swift first screening. The small difference between validation and test accuracies of all models demonstrates proper generalization and decreases the risk of overfitting, improving their potential for clinical deployment.

A further assessment (Table 2)with finer-grained details of per-class precision, recall, and F1-score, along with fold-wise accuracy, is provided to provide classification stability. Now, having evaluated these models, the evaluation results are as follows.

The Random Forest and Decision Tree had precision and recall parameters above 0.96 in both Alzheimer and non-Alzheimer classes and therefore fulfilled the requirement of being balanced in their sensitivity and specificity. They were able to maintain a k-fold accuracy of over 97% in each of the folds, indicating robustness to variance in the data partition. SVM maintained the same values with a marginally lower recall on Alzheimer case expression, and KNN had high precision in the non-Alzheimer cases but a lower sensitivity in identifying positive cases. These findings support the appropriateness of using ensemble-based models in critical tasks, where missing a positive case may have severe consequences, and indicate that the models are clinically generalizable.

We took into account robustness metrics for every class, in addition to accuracy, to evaluate the models’ overall performance. We performed 10-fold cross-validation for each split of the machine learning models (Random Forest, Decision Tree, SVM, KNN), and the results showed consistency between splits, with average accuracies ranging from 0.7277 (KNN) to 0.9793 (RF). Additionally, we computed AUC, sensitivity, and specificity, demonstrating balanced and dependable detection at every stage of Alzheimer’s disease. By providing constant and balanced detection of Alzheimer’s disease, the machine learning models demonstrated their dependability.

A visual representation of the true and false classifications of the different models reveals clear variations in error patterns. For such visualization, the confusion matrix of ML models is provided.

**Figure 6:**
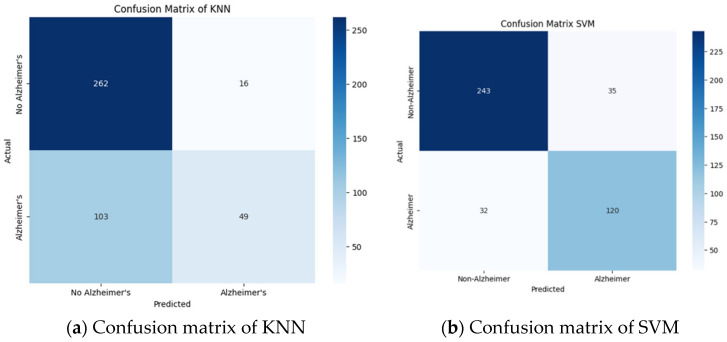

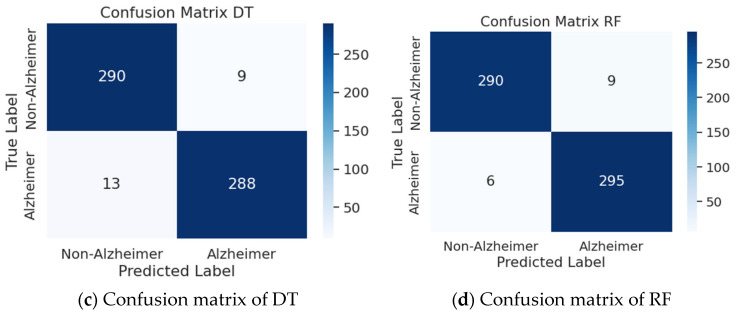
Confusion matrices of ML models (**a**) KNN, (**b**) SVM, (**c**) Decision Tree, and (**d**) Random Forest models, displaying their performance in AD classification.

Random Forest (Figure 6d) and Decision Tree (Figure 6c) achieved almost perfect predictions, with few false negatives, something that is essential for making the correct diagnosis. SVM (Figure 6b) showed fair performance but with an abnormally high false-positive rate relative to tree-based models, which may cause an unwarranted imaging follow-up. KNN (Figure 6a) failed to classify many Alzheimer-positive cases, implying that it has lower sensitivity in the initial stages of the disease, where the indications overlap to a large extent with normal aging.

An insightful multi-view analysis of the trends in model accuracy, stability in learning, and separable classification is provided as follows.

**Figure 7:**
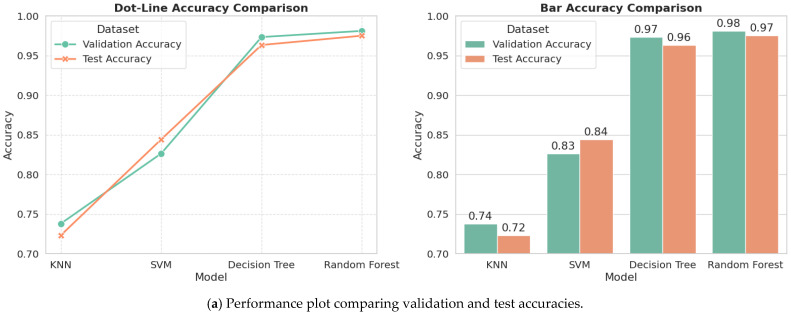

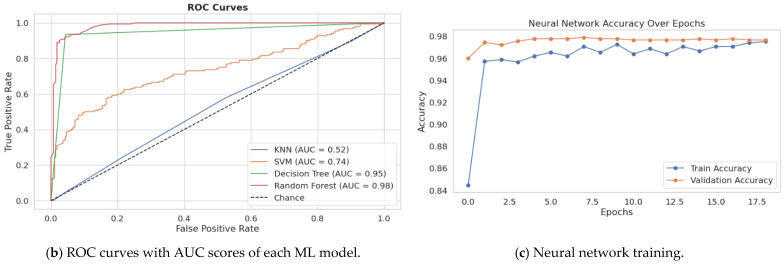
Performance plots comparing (**a**) validation and test accuracies. (**b**) ROC curves with AUC scores of each ML model. (**c**) Neural network training/validation accuracy trajectories.

Accuracy plots (Figure 7a) further confirm the results, showing the superiority of tree-based models, where there is very little decline in validation and test scores. The training curve (Figure 7b) shows quick learning, achieving 98 percent accuracy within a few cycles, which is a sign of effective learning and high power in representing features. Robustness is further confirmed using ROC curves (Figure 7c), where Random Forest and Decision Tree produced AUCs of 0.98 and 0.95, respectively, an indication of near perfection. The AUC of 0.74 for SVM suggests moderate discriminability, whereas the 0.52 of KNN is close to random chance, which underlines the utility of sophisticated classifiers in mild symptom discrimination.

To interpret which clinical features contribute most to Alzheimer’s classification, SHAP is used. The SHAP output and features for AD are as follows.

**Figure 8:**
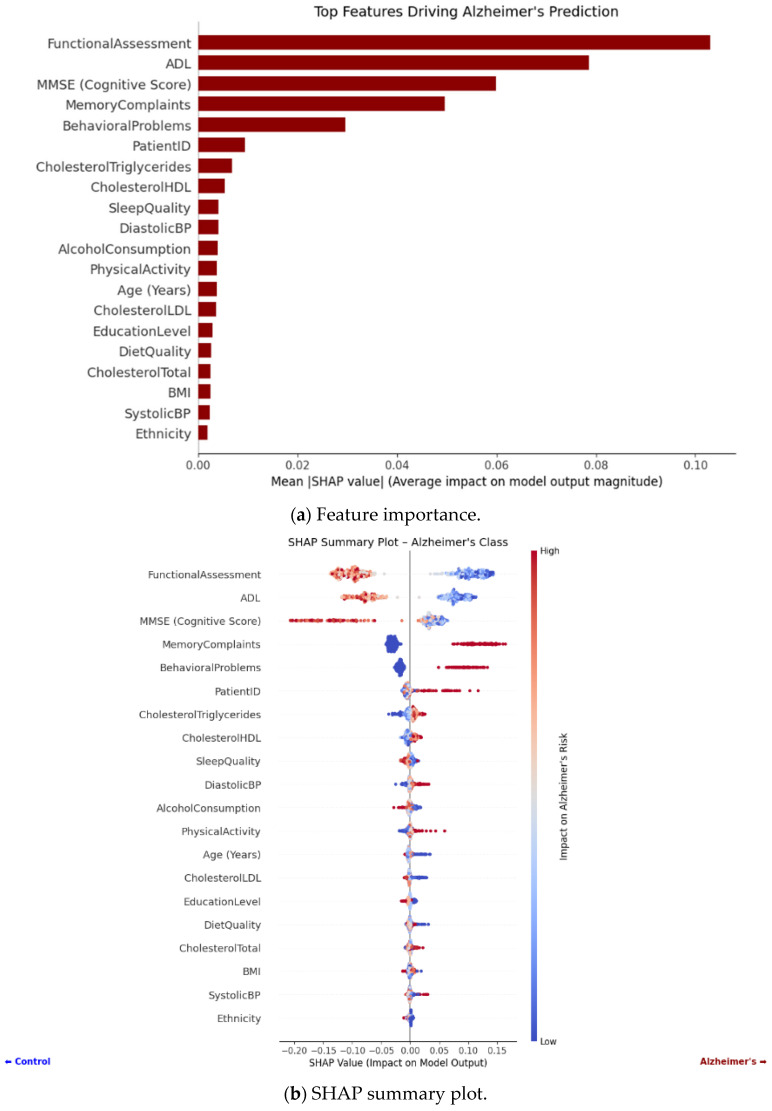
SHAP-based explainability outputs: (**a**) Model feature importance in mean absolute SHAP values, (**b**) SHAP summary plot to demonstrate the features’ influence on predictions in terms of direction and magnitude.

SHAP values were calculated with the Random Forest model trained to have a better understanding of the feature-level contributions to the Alzheimer classification task. The SHAP dot plot (Figure 8a,b) shows the contribution of each feature to an individual prediction. Dots that are colored red depict the cases where the value of the feature is high, and blue dots depict cases where the value is low. Highlighting all the dots on the *x*-axis (SHAP value) shows whether the feature took the model to the left or right of Alzheimer’s (or control). For example, features such as high age and low MMSE scores showed strong positive SHAP values, which confirms their correlation with the risk of having Alzheimer’s. The opposite case was witnessed with higher education levels and normalized brain volumes (nWBVs), which possess protective effects and were associated with negative SHAP values in most cases. Such interpretability assists in the validation of the model’s decision with clinical experience. According to the SHAP bar plot (Figure 8a), Functional Assessment, ADL, MMSE score, and memory complaints emerged as the most important predictors and correspond with diagnostic criteria. The plot with detailed outcomes (Figure 8b) demonstrates that lower cognitive and functional scores (red points) lead to Alzheimer-positive predictions, whereas high values for the scores (blue points) lead to non-Alzheimer predictions. Smaller and significant contributions of lifestyle and metabolic indicators confirm the capacity of the model to combine multi-dimensional symptom data. This transparency is a way to gain the clinician’s trust because the logic behind the model remains consistent with natural medical reasoning, while finding deeper underlying trends that would otherwise be overlooked during manual assessment.

Considering every feature from the Random Forest model used to predict Alzheimer’s disease, the table (Table 3.) below displays quantitative SHAP statistics (mean, absolute mean, Max, Min, Std).

The contributions of each clinical and demographic variable to the Random Forest model’s Alzheimer’s disease prediction are broken down feature-wise by the quantitative SHAP analysis. In comparison to the model’s baseline, SHAP values show how much each feature pushes the prediction toward Alzheimer’s (positive values) or the control (negative values). Features like Functional Assessment, ADL, and MMSE have the greatest absolute mean SHAP values in the table, indicating their predominant involvement in defining Alzheimer’s risk, but lifestyle and demographic factors like Smoking have less bearing. In order to capture the size and variability of each feature’s contribution, the table also displays the mean, absolute mean, maximum, minimum, and standard deviation of SHAP values across all patients. This thorough quantification supports the model’s interpretability in a therapeutically relevant setting and enables direct comparison of feature relevance.

Now, the effectiveness of deep learning models in MRI classification of Alzheimer’s disease stages has been tested and is reported as follows. The overall accuracy of the final tests on all the deep learning architectures used in this work was evaluated. Accuracy is defined by the number of MRI images that were accurately categorized into the corresponding stages of Alzheimer’s on the unseen test dataset. The performance Table 4 of the DL models are as follows.

The machine learning results were subjected to a one-way Analysis of Variance (ANOVA) test to see whether observed differences among models were statistically significant. In addition to reporting the F-value (ratio of between-group variance to within-group variance) and the *p*-value (probability that the observed differences occurred due to chance), ANOVA evaluates the mean performance across many models. In general, a *p* < 0.05 is regarded as statistically significant. Accuracy from the 10-tupling cross-validations was applied to the clinical-based ML models. The Random Forest outperforms KNN, SVM, and Decision Tree by a significant margin (ANOVA F = 16.94, *p* = 0.000000499 (<0.001)). ANOVA was not used for the MRI-based DL designs because of computing constraints. Instead, the same data partitions and hyperparameters were used to train and assess each deep learning (DL) model. In every run, CNN had the best accuracy, which was valid and equivalent in terms of methodology. This result shows that the DL model comparisons were not altered under different criteria and that the ML model’s superiority is supported by statistical evidence.

An ablation experiment was carried out in order to quantitatively evaluate each data modality’s independent contribution. Initially, conventional machine learning models (KNN, SVM, Random Forest, and Decision Tree) were used to assess the clinical dataset. With an average accuracy of 97%, these clinical-only models demonstrate the high discriminative usefulness of symptom-based variables for Alzheimer’s categorization. Second, deep learning models (CNN, EfficientNet-B3, ResNet-50, DenseNet-121, and MobileNetV2) were used to independently assess the MRI dataset. With an average accuracy of almost 94%, these MRI-only models demonstrated that structural brain alterations recorded in MRI scans also include significant predictive information. The Section 4 discusses the potential improvement from merging both modalities as a constraint and a direction for future study.

The comparison Table 5 between previous studies is given as follows.

The findings of the proposed dual-modal framework are contrasted with current cutting-edge techniques for classifying Alzheimer’s disease. According to Table 4, the MHAGuideNet architecture [51], 2024, as a hybrid of a pretrained 3D CNN paired with a 2D CNN and Swin Transformer, achieved an accuracy of 97.58% on ADNI and 96.02% on OASIS, while the ViT model [52], 2024, obtained an accuracy of 94% on the ADNI dataset. Utilizing ADD and OASIS datasets, our system, which uses the CNN for MRI-based stage classification and uses RF and other models for symptom-based data for the detection of Alzheimer’s disease, achieved an accuracy of 94% for the DL component and 97% for the ML component. Furthermore, our method improves interpretability, using Grad-CAM for CNN and SHAP for RF, whereas the MHAGuideNet makes no mention of explainability techniques. This shows that, in comparison to existing SOTA models, our approach provides competitive performance with enhanced explainability. Accuracy values are presented according to the original publications, but dataset configurations vary among the studies.

Furthermore, only the best-scoring deep learning models were evaluated by comprehensive assessment metrics (precision, recall, F1-score, and Jaccard Index) based on their performance at the detailed clinical level. Overall accuracy was comparable to the comparative benchmarks of other models.

A microscopic step-up survey (Table 6) of model accomplishment at every stage of Alzheimer’s. Precision quantifies how many correct positive cases were identified, and recall quantifies how many real positives were correctly picked, whereas the F1-score is their harmonic mean. The Jaccard Index takes into account overlap in predicted and actual labels, and per-class sensitivity figures denote the capacity of the model to identify each phase.

CNN performance results are well balanced across classes, with the highest recall being 97.58% versus non-dementia, with the F1-scores being high—above 0.94 in three out of four categories. DenseNet-121 also had stable results, but its Jaccard’s Index for moderate dementia (0.6846) indicates trouble differentiating this phase. EfficientNet-B3 had the highest recall (99.78) for non-dementia, and a smaller recall (55.10) for moderate dementia. This deterioration is clinically justifiable since the MRI characteristics of moderate dementia tend to overlap with both mild and advanced dementia; therefore, separating the categories is not easy. Sensitivity may also diminish as a result of the class imbalance in the available datasets. In spite of this, the clinical relevance of detection is shown by F1-scores higher than 0.70.

We took into account robustness metrics for every class, in addition to accuracy, to evaluate the models’ overall performance. We showed the diagnostic power of deep learning models (CNN, EfficientNet-b3, DenseNet-121), with each deep learning model’s Jaccard Index showing values between 0.55 and 0.93 and individual-class sensitivities between 0.55 and 0.98. This implies that while deep learning models show exceptionally high class-wise separability and robustness, exhibiting dependable detection across all phases of Alzheimer’s disease, machine learning methods also perform well. When combined, fold-wise accuracy, AUC, sensitivity, specificity, and the Jaccard Index demonstrate the reliable, broadly applicable performance of our proposed techniques. Despite exceptional class-wise independence, deep learning models excelled in accurate stage-specific Alzheimer’s identification.

**Figure 9:**
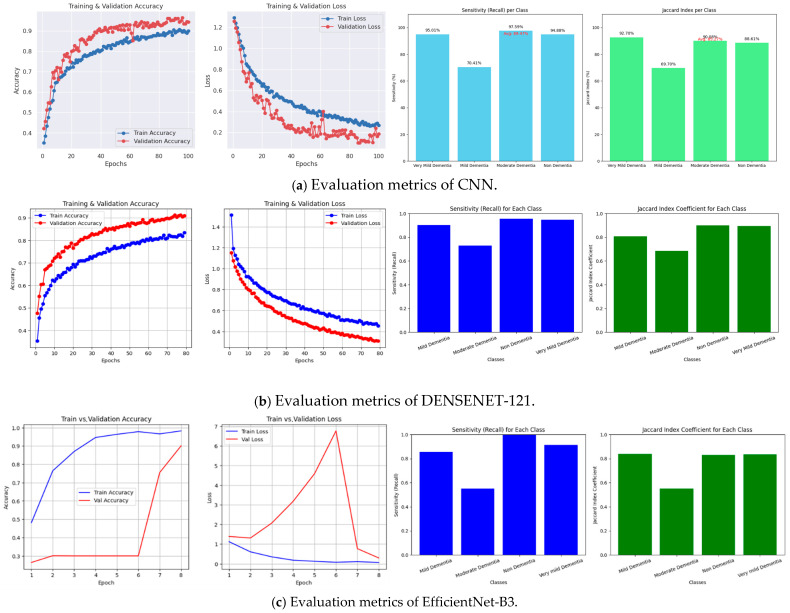
DL Model test curves and individual-class performance statistics of (**a**) CNN, (**b**) DenseNet-121, and (**c**) EfficientNet-B3. The first plot indicates training vs. validation accuracy, the second is training vs. validation loss, the third plot is per-class sensitivity (recall), and the fourth one is per-class values of Jaccard Index.

The learning stability and per-class performance of the three best deep learning models were analyzed in Figure 9a–c. Accuracy and loss plots indicate convergence characteristics concerning epochs, whereas sensitivity and Jaccard Index graphs indicate the quality status of classification per Alzheimer’s stage. Training and validation curves were tracked during the learning process in order to evaluate the models’ learning dynamics.

The CNN attained stable convergence without significant overfitting, with consistently high values of recall and Jaccard Index. Dense connectivity allowed denseNet-121 to achieve steady improvement and balanced per-class performance. EfficientNet-B3 performed well in non-dementia detection but showed a significant loss in moderate dementia sensitivity, reflecting the performance in Table 4. This is probably because of the anatomical overlap between the moderate and neighboring stages, possibly blurring the decision boundaries, and also the presence of class imbalance within the data.

The confusion matrices of the best-performing models demonstrate that correct classifications prevail, but misclassifications occur.

**Figure 10:**
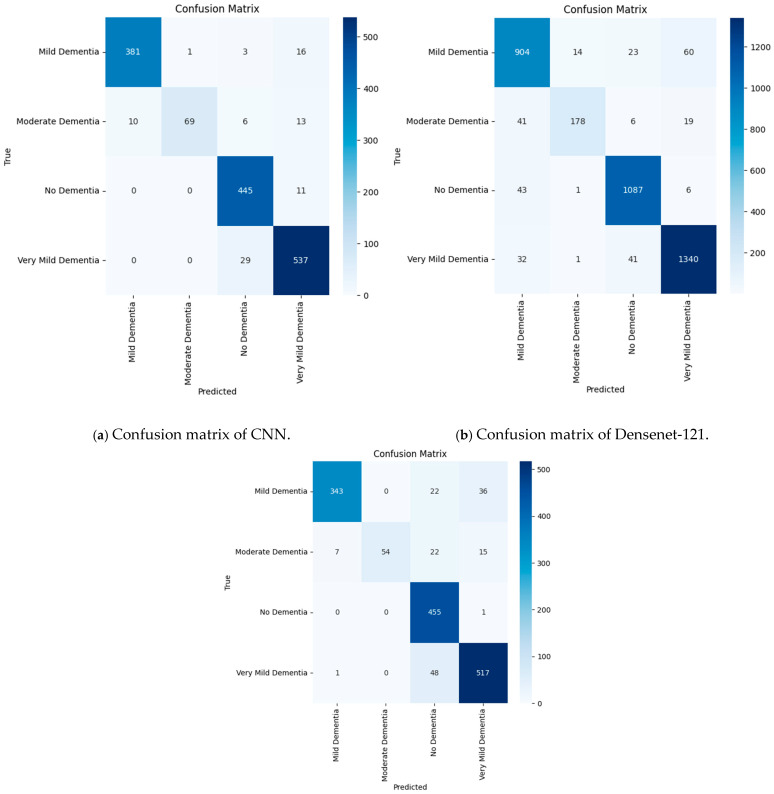
Confusion matrix of Alzheimer stage classification: (**a**) Model CNN, (**b**) DENSENET-121, (**c**) EFFICIENT-B3. These matrices show the classification performance for four categories, namely, mild dementia, moderate dementia, no-dementia, and very mild dementia. Scores on the diagonal represent correct predictions, and off-diagonal scores represent a misclassification.

Figure 10 illustrates confusion matrices of (a) CNN, (b) EfficientNet-B3, and (c) ResNet-50, considering the problem of Alzheimer’s stage. With 381 right predictions in mild dementia, 69 in moderate dementia, 465 in non-dementia, and 537 in very mild dementia, the CNN achieved significant results, whereas some of the zero scores indicate that it did not suffer from significant misclassification. EfficientNet-B3 had similar results to the CNN, with 1064 correct predictions in the cases of mild dementia, 178 in moderate dementia, 1089 in non-dementia, and 1349 in very mild dementia, with few cases of overlap between classes. ResNet-50 had a comparatively low performance, with 363 correct predictions of mild dementia, 22 of moderate dementia, 405 of non-dementia, and 517 of very mild dementia, but it was also missing some critical errors, as evidenced by the zeroes. In sum, all the models demonstrated substantial reliability in their classification, but the CNN demonstrated the best accuracy and, therefore, was chosen as the model that worked most effectively in its evaluation, which is confirmed by the Grad-CAM analysis of the CNN.

The deep learning models are interpreted by using Grad-CAM visualizations according to their predictions. These heatmaps overlay model attention on MRI scans, and they visualize which areas of the brain were the most relevant in identification.

**Figure 11:**
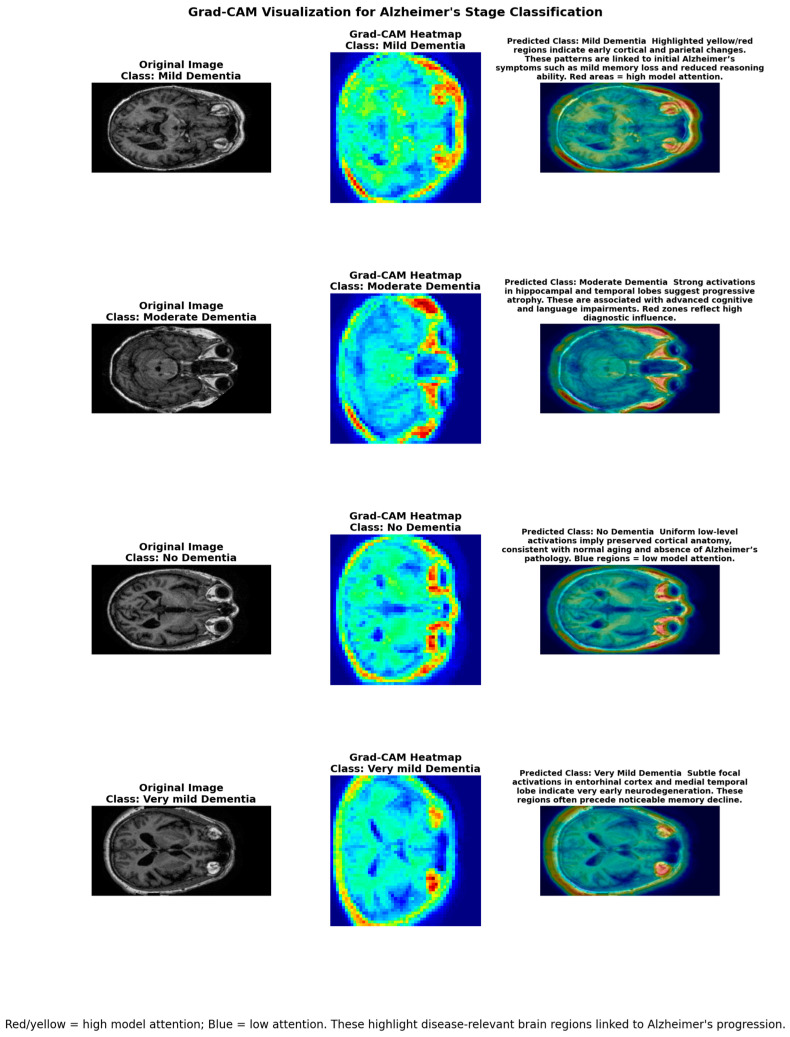
Grad-CAM heatmaps of the discriminative brain regions during the classification of the stage of Alzheimer’s. The red/yellow highlights reflect that there was much attention from the model, and the blue shows that there was little attention from the model. The activation patterns have been observed according to the clinical expectations of the disease progression.

The Grad-CAM output visualizations in Figure 11 present interpretability information of the proposed CNN on sample MRI scans from a large-scale 7000-image dataset. The heatmaps illustrate how the model probes disease-specific structural modifications in each of the classes, in line with the documented clinical variations. In mild dementia, there are high activations in cortical and parietal areas, which indicate early atrophy related to mild cognitive impairment. Moderate dementia exhibits more intensity towards the hippocampal and temporal lobes, as would be the case with progressive neurodegeneration and advanced cognitive loss, but would also occur with backward overlapping. Modest focal activations measured in the entorhinal cortex and medial temporal lobe are observed in very mild dementia; these are well-known early biomarkers of Alzheimer’s disease. In comparison, normal MRI scans demonstrate strong, homogeneous, low-level activations of the cortex, which means that the brain structure has remained intact during normal aging. The decision of the model is further attributed to the color distribution: the red/yellow areas identify the locations of high levels of diagnostic influence, and blue areas denote low contribution. The validity and interpretability of the proposed approach are supported by this close match between the areas of focus and attention to the areas of neurodegenerative patterns that are evaluated clinically.

Furthermore, the quantitative results of Grad-CAM are reported below Table 7.

**Figure 12:**
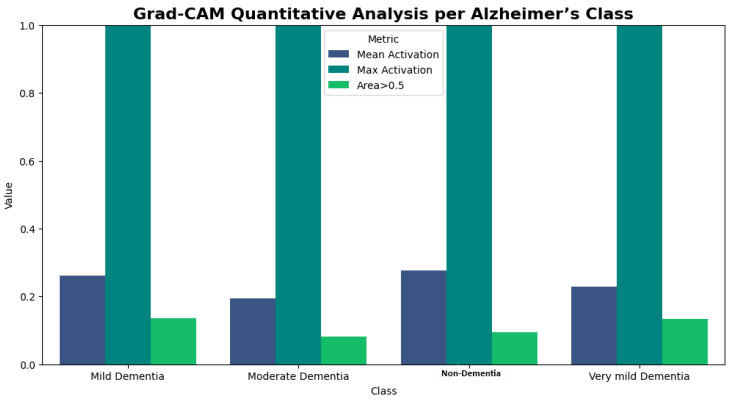
Bar plot of Grad-CAM metrics (mean, Max, Std Dev) showing class-specific attention across Alzheimer’s stages.

As shown in the above Table 7, and Figure 12 quantitatively, the regions of MRI images that the model focuses on most when categorizing Alzheimer’s staging were detected and quantified using Grad-CAM. Table 7 shows the mean activation (MA), max activation (MX), and standard deviation (SD) for every class. The mean activation shows the average amount of attention given to the highlighted areas and the degree to which the model was concentrated on recognizing class-specific features. The model continuously focused on the regions of normal brain structures during the scans, as seen by the non-dementia class’s greater mean activation (0.276). The model was concentrated locally on regions of structural degradation brought on by Alzheimer’s disease in the class of moderate dementia, as indicated by the lower mean activation (0.193). Max activation, which was the same for all classes (1.0), indicates the location of the maximum or peak attention or, to put it another way, the area with the highest level of attention in comparison to the other areas in the area. These metrics offer quantitative evidence that the Grad-CAM maps specifically target brain regions that are thought to be class-discriminative (hippocampus and cortical regions in dementia classes vs. normal regions in non-dementia cases). This shows that the model focuses on biologically significant features rather than non-biologically relevant regions. Confidence in the model’s predictions is increased when both quantitative measures and visual maps are provided.

The findings demonstrate the importance of integrating machine learning on symptom-based data and deep learning on MRI images in AD detection. Random Forest was the most accurate, with an accuracy of 97%, closely followed by Decision Tree, which achieved an accuracy of 96%. This demonstrates their performance in capturing non-labeled data based on clinical characteristics. Conversely, CNN demonstrated a high stage-wise performance (94%) among deep learning models, which demonstrated its effectiveness in detecting the nuances of neuroanatomical patterning on the basis of the images of MRI scans. These findings were further confirmed by the integration of SHAP and Grad-CAM, which showed that the model focused on biomarkers clinically used, like MMSE scores and hippocampal atrophy. The above findings support the findings that Random Forest yields optimal clinical screening, whereas CNN-based imaging is highly efficient and predictable and easily explainable in diagnosing multi-stage AD.

The most common form of dementia in the world is AD, which is clinically defined by the gradual loss of memory, lack of control, and impairment in cognition. Previous neuroimaging reports have indicated that hippocampal atrophy and cortical thinning, as well as ventricular enlargement, are characteristic signs of AD development. Furthermore, across the spectrum of Alzheimer’s disease, longitudinal data have shown shrinkage in the hippocampus and temporal cortical regions. The biological validity of stage-based predictions in our dual-modal AI paradigm is strengthened by longitudinal studies with multi-year assessments, which demonstrate that these structural alterations are probably progressive and predictive of cognitive decline. Longitudinal data improves our model’s interpretability and supports the therapeutic efficacy of MRI and symptom fusion techniques. The latest developments in AI, especially DL and ML techniques, have significantly enhanced the diagnosis and staging of AD [1,2,3,7,10].

Although most of the literature has been dominated by unimodal classification using MRI techniques or investigated only symptom-based alternatives, no studies have combined the two data types into a single framework. In addition, the issue of interpretability has not been seriously considered, but it is important, as clinicians are not sure how predictions are made. To fill these missing parts, this paper proposes a two-mode diagnostic model that includes clinical symptom data with a structural MRI scan, as well as explainable AI (SHAP and Grad-CAM), to make both reliable and true AD classifications. The main goal was to improve the early detection and stage-based classification and guarantee transparency and clinical usability [21,27,43].

Our machine learning experiments on clinical data have indicated that Random Forest achieves the highest accuracy of 97% when compared with KNN, SVM, and Decision Tree. These findings are deemed consistent, which highlights the application of ensemble approaches as particularly convenient in the diagnosis of clinical features. Convolutional neural networks that were trained using MRI images demonstrated that a designed CNN was better, with an accuracy of 94% in stage-wise classification. This means that when preprocessing and augmentation are applied, lightweight and regularized models can compete in terms of performance. Importantly, explainability analyses assisted in improving the clinical context of results. The pertinence of MMSE, age, and memory complaints as predictors of AD based on SHAP values in the clinical mode is in line with other neuropsychological studies performed in the past [28,29]. The Grad-CAM visualizations could locate the regions in the hippocampus and the ventricles, which are consistent with the regions of neurodegeneration that have been previously observed in MRI studies [14,26]. This interpretability helps fill the gap between the forecasts of AI and familiar medical knowledge.

The use of non-imaging clinical features in our dual-modal framework was highly accurate and yields better results than other prior studies with MRI alone using deep learning, indicating the value addition. Contrary to previous methods using black-box CNN models, the proposed framework incorporates XAI techniques that directly respond to the concerns mentioned by clinicians and researchers [7]. In line with the current multimodal fusion methods, our findings reveal that the integration of imaging and non-imaging biomarkers enhances strength and minimizes misclassification. In addition, our results correspond with patient-centered approaches, with the view that multimodal and interpretable pipelines may be essential in the process of promoting precision medicine for AD [32].

Although the results are encouraging, several limitations are identified. To begin with, the clinical sample targeted in this research appeared to be relatively small in comparison with large MRI datasets, which may limit the generalizability. Second, Kaggle and Google Colab offered sufficiently powerful computing resources to train the model; however, better optimization of hyperparameters is possible with high-performance clusters. Third, the framework was also tested using cross-sectional data, although longitudinal datasets would be more helpful in measuring disease progression. In future scenarios, multimodal pipelines can offer stronger diagnostic potential by incorporating other modalities, like PET images, genetic information, or even EEG responses. Further development of interpretable architectures is also expected to give clinicians even more understandable insights into the decision-making processes. The framework’s anatomical and biological significance was further supported by the explainability evaluation. Hippocampal and ventricular atrophy, two well-known neurodegenerative indicators of Alzheimer’s disease, were consistently indicated by Grad-CAM visualizations. In order to verify this result, we performed a quantitative overlap evaluation between the hippocampus ROI masks based on the ADNI atlas and the Grad-CAM activation region (top 30% intensity threshold). The conclusion that the network found structures that are clinically relevant and not spurious is supported by this analysis. The model’s interpretability and biological plausibility are supported by the near geographic match to recognized biomarkers, despite the lack of formal validation by neuroradiologists. To further improve clinical usefulness, we will build on the current work in the future by incorporating expert radiological confirmation.

The OASIS-3 dataset was used to build the framework and verify its validity; future iterations will investigate generalizability to additional datasets, such as ADNI. To reduce scanner and site variations, MRI scans were preprocessed using Z-score normalization, histogram alignment, and data augmentation. Statistical harmonization techniques will be used as part of the strategy to increase reproducibility and account for site variability. See the Section 3 for further details on the datasets. All of the datasets are publicly available and can be used by other researchers to confirm or replicate the results of this study. Though the results are encouraging, several limitations remain. The clinical sample is relatively small, and only cross-sectional data were used; longitudinal datasets would better capture disease progression. High-performance computing could further optimize hyperparameters. Future work should focus on large-scale, multi-center validation to ensure real-world applicability of this dual-modal Alzheimer’s detection framework. The direction of future work must be toward large-scale and multi-center clinical validation that would guarantee the applicability and implementation of this work to the real world.

## 5. Conclusions

This research presented a two-mode Alzheimer classification strategy, using both the machine learning and deep learning pipelines to use both clinical symptom data and MRI-based neuroimaging information. Combining these complementary modalities overcame limitations of single-source approaches, providing robust and clinically interpretable diagnostic outcomes. Random Forest and Decision Tree had the highest performance among the ML models studied, with their performance exceeding 96%, hence emphasizing that rule-based and ensemble-based classifiers are preferable in symptom-driven diagnosis. These models offered an efficient screening method that is non-invasive as a first-line screening procedure, which is very important for the detection of disease at an early stage. In the case of MRI-data-based stage-wise detection, since the ranges between normal, very mild, mild, and moderate classes of dementia were balanced, the maximum accuracy and the most balanced performance in terms of accuracy and recall were found to be provided by the CNN architecture. There were also promising results on the dense model DenseNet-121 and the efficient model EffiNetB3, but sensitivity for moderate dementia was more difficult to achieve because of the similarities in structural biomarkers. Notably, explainability methods like SHAP and Grad-CAM verified that the models were concentrating on clinically accepted characteristics, including MMSE scores, recollection issues, hippocampal reduction, and ventricular enlargement, and filled gaps between artificial intelligence predictions and medical judgment. The innovation of this work was the combination of the dual-modal data and interpretability mechanism, where not only was the classification accuracy improved, but the opinion could be trusted by clinicians and is applicable in the real environment. However, there are still shortcomings when it comes to the diversity of the dataset and the presence of class imbalances regarding mild and moderate cases of dementia. Future efforts ought to generate bigger multimodal datasets, include tracking of longitudinal progression, and investigate lightweight architectures for real-time clinical deployment. In general, the proposed framework showed that multimodal data in combination with explainable AI presents a fertile field for achieving more accurate, explicable, and clinically applicable AD diagnosis.

## Figures and Tables

**Table 1:** Test and validation accuracies of four traditional ML models being trained on symptom-based clinical data.

Model	Validation Accuracy	Test Accuracy
KNN	0.73	0.72
SVM	0.82	0.84
Decision Tree	0.97	0.96
Random Forest	0.98	0.97

**Table 2:** Comparison metrics with individual values of precision, recall, F1-score, and fold cross-validation accuracy for each of the models in AD classification.

Model	Evaluation Matrix and Its Results				K-Cross-Validation
**KNN**	Class	Precision	Recall	F1-Score	Fold-wise accuracy scores: [0.74418605 0.75813953 0.74883721 0.71627907 0.71162791 0.76744186 0.74883721 0.73953488 0.72093023 0.62149533] **Average accuracy: 0.7277**
	0	0.72	0.94	0.81	
	1	0.75	0.32	0.45	
**SVM**	Class	Precision	Recall	F1-Score	Fold-wise accuracy scores: [0.85116279 0.89767442 0.83255814 0.86046512 0.84651163 0.86511628 0.89302326 0.86976744 0.83255814 0.61682243] **Average accuracy: 0.8366**
	0	0.88	0.87	0.88	
	1	0.77	0.79	0.78	
**Decision Tree**	Class	Precision	Recall	F1-Score	Fold-wise accuracy scores: [0.98604651 0.98139535 0.97209302 0.97209302 0.97209302 0.97674419 1.0 0.97674419 0.92093023 0.52336449] **Average accuracy: 0.9282** **Average accuracy: 0.9763**
	0	0.96	0.97	0.96	
	1	0.97	0.96	0.96	
**Random Forest**	Class	Precision	Recall	F1-Score	Fold-wise accuracy scores: [0.98 0.97333333 0.98333333 0.99333333 0.98 0.97 0.98333333 0.98 0.98 0.97] **Average accuracy: 0.9793**
	0	0.98	0.97	0.97	
	1	0.97	0.98	0.98	

**Table 3:** Quantitative SHAP results that illustrate how each clinical and demographic characteristic contributes to the prediction of Alzheimer’s disease. For ease of interpretation, the mean, absolute mean, maximum, minimum, and standard deviation values are presented.

Feature	Mean–SHAP	Abs-Mean-SHAP	Max-SHAP	Min-SHAP	Std-SHAP
**Functional Assessment**	−0.005793	0.102926	0.143164	−0.138033	0.104927
**ADL**	0.009028	0.078579	0.114005	−0.118275	0.079962
**MMSE**	0.000642	0.059936	0.064907	−0.206777	0.075427
**Memory Complaints**	0.000715	0.049526	0.164452	−0.041576	0.062015
**Behavioral Problems**	−0.000289	0.029557	0.133415	−0.02697	0.041202
**Patient ID**	−0.000281	0.009367	0.11739	−0.014396	0.017682
**Cholesterol Triglycerides**	0.000417	0.006801	0.025458	−0.037017	0.008936
**Cholesterol HDL**	−0.000237	0.005295	0.019359	−0.016473	0.006316
**Sleep Quality**	−0.0007	0.00408	0.014544	−0.01645	0.005266
**Diastolic BP**	0.000466	0.004027	0.032163	−0.025018	0.006428
**Alcohol Consumption**	0.000889	0.003916	0.018117	−0.028262	0.005529
**Physical Activity**	−0.000312	0.003774	0.059548	−0.01847	0.006433
**Age**	−0.00025	0.003765	0.034379	−0.009163	0.005982
**Cholesterol** **LDL**	0.000538	0.003581	0.028318	−0.010113	0.006223
**Education Level**	−7.5 × 10^−5^	0.00281	0.010997	−0.015065	0.003969
**Diet Quality**	9.3 × 10^−5^	0.002544	0.03142	−0.007304	0.004101
**Cholesterol Total**	0.000137	0.002448	0.022069	−0.008881	0.003623
**BMI**	0.000365	0.002412	0.019106	−0.012713	0.003301
**Systolic BP**	0.000434	0.00232	0.030496	−0.007452	0.00404
**Ethnicity**	5.6 × 10^−5^	0.001891	0.005766	−0.01105	0.002384
**Disorientation**	−0.000291	0.001447	0.002238	−0.013101	0.002456
**Gender**	2 × 10^−6^	0.00113	0.004133	−0.005107	0.001382
**Diabetes**	0.000213	0.001081	0.002071	−0.00802	0.001621
**Family History of Alzheimer’s**	−9.6 × 10^−5^	0.000858	0.002389	−0.00638	0.001323
**Hypertension**	−2.1 × 10^−5^	0.000805	0.007529	−0.002377	0.001344
**Head Injury**	−4.2 × 10^−5^	0.000357	0.00526	−0.00293	0.000696
**Cardiovascular Disease**	−5 × 10^−6^	0.000607	0.006623	−0.00193	0.001048
**Depression**	−3.3 × 10^−5^	0.000483	0.004188	−0.002409	0.000734
**Confusion**	4.3 × 10^−5^	0.00048	0.005053	−0.003003	0.000809
**Personality Changes**	5 × 10^−6^	0.000726	0.003453	−0.006301	0.001213
**Difficulty Completing Tasks**	−7.6 × 10^−5^	0.000429	0.004014	−0.005451	0.000788
**Forgetfulness**	−3.2 × 10^−5^	0.0002	0.003773	−0.001689	0.000363
**Smoking**	−2 × 10^−5^	0.000133	0.001597	−0.002326	0.00028

**Table 4:** The accuracy of five deep learning architectures in the classification of AD stages was evaluated.

Model	Test Accuracy
CNN	0.94
DenseNet-121	0.9244
EfficientNet-B3	0.9020
ResNet-50	0.7219
MobileNetV2	0.7033

**Table 5:** The proposed dual-modal ML (RF) + DL (CNN) architecture is compared with previous AD classification models in terms of accuracy and explainability. In the case of explainability for MHAGuideNet, none is reported because no explainability methods were used.

Study	Dataset	Method	Accuracy	Explainability
**ViT (2024)**	ADNI	Vision Transformer	94%	Grad-CAM
**MHAGuideNet (2024)**	ADNI + OASIS	Hybrid Model (Pretrained 3D CNN + 3D CNN + Swin Transformer)	97.58% (ADNI), 96.02% (OASIS)	None Reported
**This Study**	ADD and OASIS	Dual-Modal ML (AD Detection) and DL (Stage Classification)	97% and 94%	SHAP and Grad-CAM

**Table 6:** Detailed per-class evaluation of CNN, DenseNet-121, and EfficientNet-B3 models. Metrics include precision, recall (sensitivity), F1-score, Jaccard Index coefficient, and per-class sensitivity in detecting mild dementia, moderate dementia, non-dementia, and very mild dementia.

Model	Evaluation Matrix and Its Results				Jaccard Index Coefficient for Each Class	Sensitivity for Each Class
**CNN**	Stage of Alzheimer’s	Precision	Recall	F1-Score	[0.9270073 0.6969697 0.90080972 0.88613861]	[0.95012469 0.70408163 0.97587719 0.94876325]
	Mild Dementia	0.97	0.95	0.96		
	Moderate Dementia	0.99	0.70	0.82		
	Non-Dementia	0.92	0.98	0.95		
	Very Mild Dementia	0.93	0.95	0.94		
**Densenet-121**	Stage of Alzheimer’s	Precision	Recall	F1-Score	[0.80931065 0.68461538 0.90057995 0.89392929]	[0.9030969 0.7295082 0.95602463 0.9476662]
	Mild Dementia	0.89	0.90	0.89		
	Moderate Dementia	0.92	0.73	0.81		
	Non-Dementia	0.94	0.96	0.95		
	Very Mild Dementia	0.94	0.95	0.94		
**Efficient-b3**	Stage of Alzheimer’s	Precision	Recall	F1-Score	[0.83863081 0.55102041 0.83029197 0.83656958]	[0.8553616 0.55102041 0.99780702 0.91342756]
	Mild Dementia	0.98	0.86	0.91		
	Moderate Dementia	0.99	0.55	0.71		
	Non-Dementia	0.83	0.99	0.91		
	Very Mild Dementia	0.91	0.91	0.91		

**Table 7:** Grad-CAM quantitative metrics (mean, Max, Std Dev) for each Alzheimer’s stage.

Class	Mean Activation	Max Activation	Std Deviation	Area > 0.5
**Mild Dementia**	0.2608	1.0	0.2248	0.1361
**Moderate Dementia**	0.1932	1.0	0.1920	0.0821
**No Dementia**	0.2762	1.0	0.2004	0.0947
**Very Mild Dementia**	0.2280	1.0	0.2330	0.1339

## Data Availability

The datasets used in this study are publicly available and can be accessed at https://www.kaggle.com/datasets/ninadaithal/imagesoasis/; https://www.kaggle.com/datasets/rabieelkharoua/alzheimers-disease-dataset (accessed on 30 September 2024). No new data were created or collected specifically for this study.

## Abbreviations

The following abbreviations are used in this manuscript:

AD	Alzheimer’s Disease
ML	Machine Learning
DL	Deep Learning
XAI	Explainable AI
